# Immunomodulatory Activity of a Traditional Sri Lankan Concoction of *Coriandrum sativum* L. and *Coscinium fenestratum* G.

**DOI:** 10.1155/2020/9715060

**Published:** 2020-09-14

**Authors:** Shashika Dinethri Kothalawala, Daniya Edward, Jayamini C. Harasgama, Loshini Ranaweera, Ovitigala Vithanage Don Sisira Jagathpriya Weerasena, Roshan Niloofa, Wanigasekera Daya Ratnasooriya, Galbada Arachchige Sirimal Premakumara, Shiroma M. Handunnetti

**Affiliations:** ^1^Institute of Biochemistry, Molecular Biology and Biotechnology (IBMBB), University of Colombo, Colombo, Sri Lanka; ^2^Department of Zoology and Environmental Science, Faculty of Science, University of Colombo, Colombo, Sri Lanka; ^3^Department of Herbal Technology, Industrial Technology Institute, Bauddaloka Mawatha, Colombo 7, Sri Lanka

## Abstract

**Objective:**

To investigate the immunomodulatory activity of a traditional Sri Lankan concoction of *Coriandrum sativum* L. and *Coscinium fenestratum* (Gaertn.) Colebr., which is a Sri Lankan traditional medicine used to relieve inflammation and cold.

**Methods:**

*In vivo* anti-inflammatory activity was tested using carrageenan-induced rat paw-edema model. Mechanism of anti-inflammatory activity was assessed by investigating the production of nitric oxide (NO), expression of iNOS enzyme, and reactive oxygen species (ROS) by rat peritoneal cells. The membrane stabilizing activity was also tested. The antibody response was determined by assessing the specific haemagglutination antibodies raised against sheep red blood cells.

**Results:**

The three doses of freeze-dried concoction used ((human equivalent dose (HED)—183 mg/kg) 2 × HED and 1/2HED; *n* = 6 rats/group) showed significant inhibition of paw edema compared to water control at 3^rd^–5^th^ hours (*p* < 0.05). Both HED and 1/2HED exhibited marked anti-inflammatory activity (72–83% inhibition at 4^th^-5^th^ hours; *p* < 0.05). The HED of the concoction showed significant inhibition of NO (77.5 ± 0.73%, *p* < 0.001) and ROS production (26.9 ± 2.55%; *p* < 0.01) by rat peritoneal cells. Inhibition of NO production in the concoction treated rat peritoneal cells was confirmed by the lack of iNOS expression. The concoction also exhibited significant membrane stabilizing activity (IC_50_ = 0.0006 *μ*g/ml; *p* = 0.001). HED resulted in a significantly high induction of specific antibody production against SRBC antigens as detected by SRBC haemagglutination assay (mean day 14 titers 253.3 compared to control: 66.7) (*p* < 0.01).

**Conclusions:**

The traditional Sri Lankan concoction of *C. sativum* and *C. fenestratum* demonstrated potent *in vivo* anti-inflammatory activity, significant reduction of ROS, and NO production by rat peritoneal cells and the lack of iNOS expression confirmed the low NO production. The increased membrane stability also supports the anti-inflammatory activity of the concoction. Further, this concoction induced a significantly high antibody response reflecting its immunostimulatory activity. Together these results scientifically validate the therapeutic use of the concoction of *C. sativum* and *C. fenestratum* in Sri Lankan traditional medicinal system for immunomodulatory effects.

## 1. Introduction

Many medicinal plants are found to have an array of pharmacological properties that could be applied in immunomodulation such as immunostimulants, tonic, neurostimulant, antibacterial, antiviral, antirheumatic, and anticancer [1]. In Sri Lanka, many herbs and medicinal plants are used in Ayurveda and in indigenous medicinal practices for centuries. In traditional medicine, the combination of *Coriandrum sativum* L. (family: Apiaceae) and *Coscinium fenestratum* (Gaertn.) Colebr. (family: Menispermaceae) is used as an immunomodulator for various types of ailments including relief of pain, inflammation, cold, and other viral infections for centuries [2]. Immunomodulatory and anti-inflammatory agents are therapeutically important since the pathogenesis of the common cold involves a complex interplay between replicating viruses and the host's inflammatory response [3].

A decoction is made using equal amounts of seeds of *C. sativum* (coriander; “*Kottamalli*” or “*Kothamburu*” in Sinhala and “*Kottamalli*” in Tamil) and stem of *C. fenestratum* (calumba wood or tree turmeric, “*Veniwalgatta*” in Sinhala; “*Maramanjal*” in Tamil) is a well-known home remedy in Sri Lanka for cold and inflammations, especially during the early stage of infection [2]. These two ingredients are also constituents of the commercially available formulations called “Paspanguwa” along with three other plants parts, *Zingiber officinale* Roscoe., *Oldenlandia corymbosa* L., and *Solanum surattense* Burm.f.) and also in another commercial formulation called Samahan which is a combination of 14 ingredients including these two [4].

The two plant parts, seeds of *C. sativum* and stem of *C. fenestratum,* are known to have a range of uses in traditional medicine and in Ayurveda. Coriander is used for treatment for anxiety, flatulence, loss of appetite, and convulsions [5]. Coriander seeds are used as carminative, diuretic, tonic, stimulant, stomachic, cooling agent, aphrodisiac, and analgesic [6]. Coriander has been attributed to have several medicinal uses, having antidiabetic, diuretic, cholesterol lowering, anticancer, anti-inflammatory, antifungal, antihelmintic, antioxidant, and antimicrobial effects [7–11]. Stem of *C. fenestratum* is thermogenic, ophthalmic, anti-inflammatory, vulnerary, depurative, stomachic, antiseptic, febrifuge, sudorific, and tonic [12, 13]. Stem pieces of *C. fenestratum* are boiled and one cup is given for a fresh, deep cut, being the most common use against tetanus [2]. The root bark is used for dressing wounds and ulcers. *C. fenestratum* powder is mixed with ghee and used to apply for quick healing of ulcers. For snake bite poisoning, paste of *C. fenestratum* and turmeric is applied. *C. fenestratum* is reported to have anticancer, antimicrobial, antidiabetic, and antioxidant effect and is also used to treat cholera, gastroenteritis, and bleeding piles [14–16]. The seeds of *C. sativum* and stem of *C. fenestratum* have previously been shown to have anti-inflammatory activity when tested alone and as ethanolic concoctions of individual ingredients [17, 18]. Some immunostimulatory activity has also been reported with aqueous and ethanolic concoctions of *C. sativum* when used as a single ingredient [19–22].

The main objective of this study was to scientifically validate the traditional use of this concoction of seeds of *C. sativum* and stem of *C. fenestratum* as an immunomodulator. More specifically, we investigated its *in vivo* anti-inflammatory activity using the carrageenan-induced rat paw-edema model and its effect on some of the immune cellular mechanisms including the production of nitric oxide (NO) and reactive oxygen species (ROS) and the expression of inducible nitric oxide synthase (iNOS) by rat peritoneal cells, membrane stabilizing activity of the concoction, and its immunostimulatory activity in enhancing antibody response.

## 2. Materials and Methods

### 2.1. Materials

All chemicals and consumables, unless otherwise stated, were purchase from Sigma Aldrich, USA. Wistar Albino rats and Sheep red blood cells (SRBC) were purchased from Medical Research Institute (MRI), Colombo 08, Sri Lanka. Rat glyceraldehyde-3-phosphate dehydrogenase (GAPDH), rat iNOS, endothelial NOS (eNOS), and neuronal NOS (nNOS) primers and random primers were obtained from Integrated DNA Technologies, USA. RT-PCR and PCR reagents including chloroform, diethyl pyrocarbonate, dNTPs, Go Taq Flexi buffer, isopropyl alcohol, MgCl_2_, M-MLV reverse transcriptase, RNasin®, RT buffer, and Taq polymerase were purchased from Promega Cooperation. Madison, USA. DNA (100 base pair) ladder was obtained from New England Bio Labs United Kingdom.

The reference drugs, aspirin, indomethacin, and prednisolone and also the syringes, needles, surgical blades, and cannulas (18G) were purchased from State Pharmaceuticals Corporation of Sri Lanka. Tissue culture plates (24 wells and 96 wells round and flat bottom), plates for Enzyme Linked Immunosorbant Assay (ELISA), round bottom tissue culture plates were purchased from Nunc, USA, and nitrocellulose filters (2 *μ*M) and Whatman filter papers (No. 1) were obtained from Whatman Int. Ltd., UK. Reusable rat feeding needle was purchased from Orchid Scientifics, India.

### 2.2. Preparation of the Concoction of *C. sativum* and *C. fenestratum*

Seeds of *C. sativum* and stems of *C. fenestratum* were purchased from a reputed Ayurvedic store in Colombo, Sri Lanka, and authenticated by Dr. Chandima Wijesiriwardena at the Industrial Technology Institute, Colombo, Sri Lanka. Voucher specimens of *C. sativum* and *C. fenestratum* were deposited at the Institute of Biochemistry, Molecular Biology, and Biotechnology (IBMBB), University of Colombo, Sri Lanka.

The concoction was made according to traditional Sri Lankan medicinal practice [2], by boiling 30 g each of *C. sativum* seeds and *C. fenestratum* stem in 1920 ml of water in a copper vessel till it reached approximately 240 ml. The concoction was filtered using Whatman No. 1 filter paper and freeze dried (Freezone 4.5-Labconco Corporation, USA). The human equivalent dose (HED) was calculated using the following formula [23].

(1) $$\begin{array}{c} \text{HED}\left(\text{mg}\right)=\frac{\text{weight of the concoction from }120\text{ ml}\left(\text{mg}\right)\times \text{weight of the rat}\left(\text{kg}\right)\times 6.2}{\text{Weight of a normal human}\left(60\text{ kg}\right)},\\ \text{Rat metabolic rate}=6.2. \end{array}$$

### 2.3. Experimental Animals

Wistar strain adult male and female rats weighing 150–250 g were purchased from the Medical Research Institute, Colombo, Sri Lanka. Rats were acclimatized for one week and randomly grouped (*n* = 6) according to their weights. Rats were housed in the animal house of IBMBB, University of Colombo, under standard conditions (temperature 28–31°C, photoperiod approximately, 12 hours natural day light per day, relative humidity 50–55%). The animals were fed with pellet food purchased from Diamond Stores, Colombo 06, Sri Lanka, and clear drinking water *ad libitum*.

All experiments were conducted in accordance with the internationally accepted laboratory animal use and care, based on 3 Rs. Ethical clearance was obtained from the Research, Ethics and Higher Degrees committee of the IBMBB, University of Colombo. Animals were subjected to mild ether anesthesia for all procedures.

### 2.4. Assessment of *In Vivo* Anti-Inflammatory Activity of the Concoction by Using Carrageenan-Induced Rat Paw-Edema Assay

Three doses of freeze-dried concoction-human equivalent dose (HED-183 mg/kg), high dose (2 × HED-366 mg/kg), and a low dose (1/2HED - 92 mg/kg), were orally administered to three groups of rats (*n* = 6/group). Indomethacin (5 mg/kg) was used as the reference drug (positive control) and water (2 ml) was administered to the control group. Paw volumes were measured hourly, after the carrageenan (0.1 ml of 1% carrageenan) injection on the left hind paw by using a digital Plethysmometer (Panlab sl., Barcelona, Spain) as described previously [24, 25].

### 2.5. Assessment for Nitric Oxide Production by Rat Peritoneal Cells

Peritoneal cells were collected as described previously [26]. Three groups of rats were orally treated with HED (the optimum dose selected), prednisolone as reference drug (10 mg/kg), and water as control. One hour after the oral treatment, 1 ml of 0.1% carrageenan (1 mg/ml) was injected to the rat peritoneal cavity. Two hours after this, 40 ml of sterile phosphate buffered saline (PBS) was injected and approximately 35 ml of fluid was drained from peritoneal cavity. The drained peritoneal fluid was centrifuged at 500*g* for 10 minutes and resuspended in 1 ml of RPMI-1640 medium containing 1% bovine serum albumin (BSA) and total cell and differential cell counts were taken using a Neubauer's haemocytometer (Neubauer, Germany).

Rat peritoneal cells as described above were used to evaluate the inhibitory effect of the HED against the production of nitric oxide. Cell suspension (200 *μ*l of 1 × 10^6^/ml) was plated in 96 well tissue culture (TC) plates where 6 wells were maintained for each rat. The TC plate was incubated for 24 hours at 37°C in a 5% CO_2_ incubator. After 24 hours, the supernatant was collected, centrifuged at 10,000*g* for 10 minutes, and stored at −20°C for quantification of nitrite levels. Griess assay was used to quantify nitrite levels in rat peritoneal culture supernatants by mixing 100 *μ*l of culture supernatant with equal volume of Griess solution (equal amounts of 1% Sulphanilamide and 0.1% *N*-(naphtyl) ethlenediamine hydrochloride) [27]. Optical density at 540 nm was measured 15 minutes after adding the Griess solution using an ELISA microplate reader (ELx 800-Universal Microplate Reader, Biotek Instruments, Canada). A dilution series of NaNO_2_ standards from 100 to 0.781 *μ*M were used to prepare nitrite standard curve. The amount of nitrites in *μ*M was computed from the standard curve plotted for NaNO_2_.

### 2.6. Assessment for the Expression of iNOS by Rat Peritoneal Cells

Total RNA was extracted from rat peritoneal phagocytic cells using the TRIzol reagent (1 ml of TRIzol to 1 × 10^6^ cells) according to the manufacturer's instructions (Invitrogen, USA). Extracted RNA was quantified and then cDNA was synthesized using RNA (2 *μ*g), dNTPs mixture (2 mM), random primers (500 ng), RNAsin (25 units), M-MLV reverse transcriptase enzyme (200 units), and the RT buffer (1X) and PCR was carried out for selected genes iNOS, eNOS, nNOS, and GAPDH independently using the same cDNA. Primers for rat iNOS were selected according to Linenluke et al. and thermal cycle parameters were initial denaturation at 94°C for 5 min followed by 35 cycles of 94°C for 1 min, 62°C for 1 min, 72°C for 1 min, and the final extension of 72°C for 10 min [28]. Primers for other constitutive forms of NOS, eNOs, and nNOS were selected as indicated in Liu et al. (annealing temperature for rat eNOS was 60°C, whereas the annealing temperature for rat nNOS was 62°C) [29]. Rat GAPDH gene which was used as the control or house-keeping gene was also amplified with the primers indicated in Wu et al. [30]. All amplified PCR products were resolved in 2% agarose gel and visualized by the UV transilluminator (Vilber–Laumart gel documentation system).

### 2.7. Assessment of ROS Production in Rat Peritoneal Cells

Peritoneal cells collected as described in Section 2.5 were used to evaluate the effect of the HED against the production of ROS. Concentration of cell suspensions were adjusted to 4 × 10^5^ cells/ml using complete RPMI containing 10% fetal bovine serum (cRPMI) and 8 × 10^4^ cells in 200 *μ*l of cell suspension were plated in 24-well culture plate and the final volume/well was increased to 400 *μ*l with 200 *μ*l of cRPMI added to each well. For each rat, 3 wells were maintained. Diphenyleneiodonium chloride (DPI) was used as *in vitro* positive control. For this, 200 *μ*l of cell suspension obtained from a rat treated with water was plated with 200 *μ*l of 10 *μ*M DPI in cRPMI. Plate was incubated for 1 hour at 37°C with 5% CO_2_ to allow the cell attachment. After one hour, 200 *μ*l of supernatant was removed, 200 *μ*l of 2 mg/ml of nitro blue tetrazolium (NBT) with 12 *μ*g/ml phorbol 12-myristate 13-acetate was added to each well and the plate was incubated for 30 minutes at 37°C with 5% CO_2_. After half an hour supernatant was removed and plate was washed twice with prewarmed (37°C) PBS. The plate was fixed using 70% methanol and allowed to dry and 120 *μ*l of 2 M KOH and 140 *μ*l of absolute dimethyl sulphoxide were added to each well and the plate was placed on a shaker for 10 minutes. Dissolved formazan (200 *μ*l) was transferred into 96-well ELISA plate and absorbance was read at 620 nm. The concentration of O_2_^−^ was calculated using standard NBT curve as described previously [31].

### 2.8. Assessment of Membrane Stabilizing Activity of Concoction

This assay was performed by heat-induced haemolysis of rat erythrocytes as described previously [26]. Ten-fold dilution series of concoction was made using PBS for concentrations from 0.0001 to 1000 *μ*g/ml. Dilutions of Aspirin was also made using PBS for the same concentrations and used as the standard drug. PBS was used as control. Rat erythrocytes washed and resuspended in PBS (20 *μ*l) was added to each tube containing 980 *μ*l of each concentration of test, aspirin and control samples. Samples were first incubated at 37°C for 15 min. Cell suspensions were centrifuged at 1500*g* for 3 min, the supernatants were removed and the cells were resuspended in 1 ml of PBS. Samples were then incubated at 54°C for 25 min to initiate heat-induced haemolysis and centrifuged at 1500*g* for 5 min. Supernatants (200 *μ*l) were transferred into an ELISA plate and the optical density (OD) was measured at 540 nm. Percentage inhibition of haemolysis was calculated with respect to the controls and inhibitory concentration (IC_50_) values were derived. Percent inhibition of haemolysis = [(OD control − OD sample/OD control] × 100.

### 2.9. Assessment of the Effect of the Concoction on Rat Antibody Production and Detection of Antibodies by Haemagglutination Test

This experiment was designed to investigate the effect of oral treatment of rats with the concoction on their specific antibody production against SRBC antigens. The SRBC immunization was performed according to a modification of the previously described method [32–34]. Two groups of rats (*n* = 6/group) were orally treated with HED of concoction and water on days 1, 2, 3, 7, 8, and 9. A preparation of 0.5 × 10^9^ cells of freeze-thawed SRBC was injected intraperitoneally on days 1 and 7. Serum collected on days 0, 7, and 14 was tested for anti-SRBC antibodies using SRBC haemagglutination assay [35]. Day 0 (preimmune) sera were used as the negative control, while days 7 and 14 sera were collected to ascertain the levels of antibodies after the exposure to SRBC antigen. Haemagglutination plates were incubated at 37°C for 16 hours.

### 2.10. Statistical Analysis

Data were analyzed using the statistical package SPSS 17. Data were expressed as mean ± SD/SEM. One-way ANOVA was carried out; *p* ≤ 0.05 were considered as significant. The Mann–Whitney *U* test and independent *t*-test were carried out for small sampled tests. One-way ANOVA followed by post hoc Turkey was carried out to compare the inhibition of *in vivo* anti-inflammatory activity. Pearson corelation was calculated for dose dependency.

## 3. Results

### 3.1. *In Vivo* Anti-Inflammatory Activity of the Concoction

**Figure 1:**
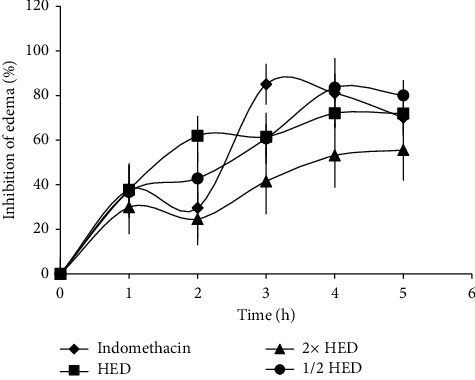
The inhibition of rat paw edema by the concoction of *C. sativum* and *C*. *fenestratum*. Inhibition of paw volume was assessed from rats treated with 1/2HED, HED and 2HED of the concoction, indomethacin (5 mg/kg), and water. HED: Human equivalent dose. Values represent mean ± SEM; *n* = 6 rats per group.

All three doses of the concoction showed significant anti-inflammatory activity which was comparable to the positive control indomethacin (Figure 1). There was a significant decrease in paw volumes in all three groups, compared to water control at 3^rd^, 4^th^, and 5^th^ hours (*p* < 0.05). The doses, 1/2HED and HED, overlapped at the first and the third hour and the ½HED showed the highest percentage inhibition in paw volumes at the fourth (83.5%) and the fifth (80.1%) hours followed by HED (72%). The percent inhibition of the double dose (2 × HED) increased gradually and reached its maximum at the 5^th^ hour (55.6%); however, its overall anti-inflammatory effect was low compared to the other two doses (1/2HED and HED). This resulted in an inverse dose-dependent activity at 4^th^ (*r* = −0.99; *p*=0.03) and 5^th^ (*r* = −1.00; *p*=0.001) hours. Since HED showed a significant level of anti-inflammatory activity in the first and second phases of inflammation, it was selected as the optimum dose for further assays. The concoction reported hereafter in the results section is the HED of the concoction.

### 3.2. Inhibition of Nitric Oxide Production and iNOS Expression by Rat Peritoneal Cells

**Figure 2:**
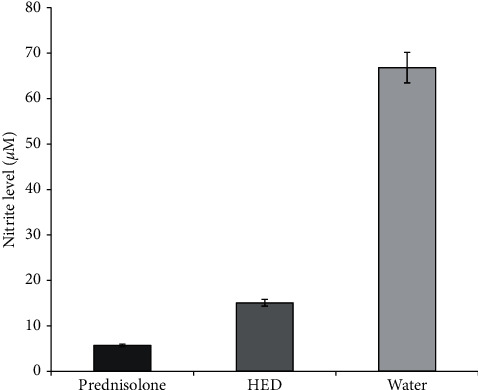
Effect of the human equivalent dose (HED) of the concoction on NO production by rat peritoneal cells. NO production by peritoneal cells obtained from rats treated with HED of the concoction and prednisolone (10 mg/ml) and from control group was assessed. Data represents mean ± SEM.

As shown in Figure 2, nitrite level was significantly inhibited by the concoction and percent inhibition was 77.5 ± 0.73% (*p* < 0.001). The reference drug and prednisolone had inhibited nitrite levels similarly by 91.5 ± 0.69% (*p* < 0.001).

**Figure 3:**
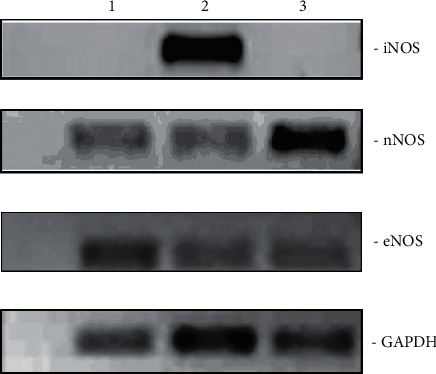
NOS gene expression in peritoneal cells from carrageenan-injected rats orally administered with prednisolone, lane 1; water, lane 2; and the concoction, lane 3. iNOS: inducible NOS; nNOS: neuronal NOS; eNOS: endothelial NOS; GAPDH: glyceraldehyde-3-phosphate dehydrogenase.

As shown in Figure 3, amplifications of GAPDH were observed in all three samples (RNA obtained from the rats injected with carrageenan and treated with the reference drug (prednisolone), water, and concoction). Amplification of iNOS was observed only in the control (water treated) and was absent in the concoction treated and the group treated with the reference drug. Amplification of nNOS was clearly observed for concoction treaded group, whereas amplification of both nNOS and eNOS was weak in the group treated with the reference drug.

### 3.3. Inhibition of *In Vivo* ROS Production by Rat Peritoneal Cells

**Figure 4:**
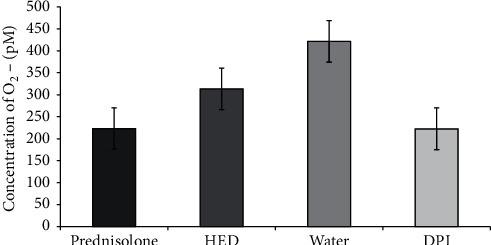
Effect of the human equivalent dose (HED) of the concoction on ROS production by rat peritoneal cells. ROS production by peritoneal phagocytic cells harvested from rats treated with HED of the concoction, prednisolone (10 mg/ml), and water as control. Cells treated with diphenyleneiodonium chloride (DPI) used as *in vitro* positive control.

As shown in Figure 4, concoction had significantly inhibited the O_2_^−^ production and percent inhibition was 26.9 ± 2.55% (*p*=0.002). The two positive controls, *in vivo* (prednisolone) and *in vitro* (DPI), had also inhibited the O_2_^−^ production significantly and percent inhibitions of O_2_^−^ production were 47.8 ± 1.78% and 48.5 ± 1.97%, respectively (*p* < 0.001).

### 3.4. Membrane Stabilizing Activity of the Concoction

**Figure 5:**
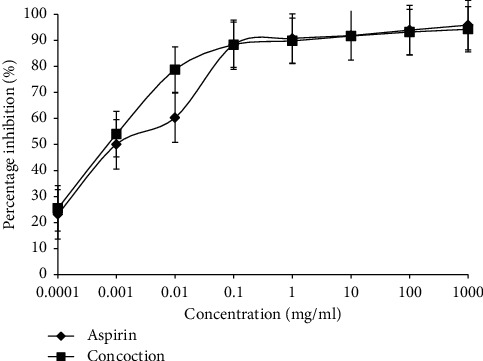
Membrane stabilizing activity of the concoction. Percent inhibition of haemolysis of rat red blood cells treated with the concoction and aspirin (positive control) was assessed. Data represent average from three repeat experiments, *n* = 6. Data represents mean ± SEM.

As shown in Figure 5, the concoction showed 88.34% of inhibition for heamolysis at 0.1 mg/ml and comparable to the reference drug aspirin (88.36%; *p*=0.001) and IC_50_ value of the concoction was 0.0006 *μ*g/ml.

### 3.5. Effect of Oral Administration of the Concoction on Rat Antibody Production

Effect of oral administration of the concoction on rat antibody production was evaluated by assessing the SRBC haemagglutination titers. The sera obtained on day 0 showed no antibodies against SRBC in both groups. On day 7, the SRBC haemagglutination titers in both concoction and control groups of rats were low and comparable (*p* > 0.05), but both groups showed agglutination (Table 1). Titers of the concoction group on days 7 and 14 increased significantly from 100 (mean) to 253.33 (*p*=0.03), whereas no significant change was observed in the control group. By day 14, the group of rats treated with the concoction showed a significant increase in their SRBC haemagglutination titers compared to day 14 sera of the control (mean value 253.3 and 66.7, respectively; *p*=0.004). This showed that after two sets of 3-day oral treatment (and with two sets of antigen exposures), the concoction was able to induce a significant increase in the specific antibody response against SRBC.

## 4. Discussion

This study was designed to determine the immunomodulatory activity of the concoction of *C. sativum* and *C. fenestratum*. This was first ascertained by the *in vivo* anti-inflammatory activity using the carrageenan-induced rat paw edema model and the results showed a significant anti-inflammatory activity of the concoction. Since HED of concoction showed a significant level of anti-inflammatory activity at both first and second phases of inflammation, it was selected as the optimum dose for subsequent experiments to determine possible mechanisms of its anti-inflammatory activity. Further, this study demonstrated significant inhibition of nitric oxide and superoxide anion production by rat peritoneal cells and also *in vitro* membrane stabilizing capacity reflecting their possible contribution to the anti-inflammatory mechanisms. The specific inhibition of the expression of iNOS by the oral administration of the concoction confirmed the marked decrease of NO production by the rat peritoneal cells. The immunomodulatory activity was also shown by the enhancing effect on specific immune responses as evident by the high antibody titers raised against SRBC antigens following oral administration of concoction. This study reports for the first time the immunomodulatory activity of the concoction of *C. sativum* and *C. fenestratum,* including *in vivo* anti-inflammatory activity with mechanisms, that is, inhibition of ROS and RNS production and also the inhibition of iNOS gene expression by rat peritoneal cells and the immunomodulation to enhance antigen specific antibody response. Further, the findings of this study validate the use of the concoction (or decoction of *C. sativum* and *C. fenestratum*) for its traditional claims and use for treatment of cold and as a treatment at early stage of infections.

In the carrageenan-induced paw-edema assay, the two doses (HED and 1/2HED) of the concoction showed significant anti-inflammatory effect during both early and late phases and there was marked inhibition during the late phase. The difference in the phases of the assay may occur due to different chemical and cellular components which come into action in the early, intermediate, and late phases of inflammation [36]. The inhibition in the first phase of inflammation is attributed to activities against serotonin and histamine, whereas inhibition during second phase is mainly due to activities against prostaglandins and suppression of mononuclear leukocyte migration [36]. In contrast to the biphasic pattern, the high inhibition during 2^nd^ hour (intermediate phase) shown by the HED of the concoction indicates that it may have inhibited the kinins which are known to play a role in between the first and second phases [37]. The inverse dose response observed in the present study with the 2 × HED exhibiting a lower anti-inflammatory activity is consistent with the results of a previous study where the potency of a high dose of an alcoholic preparation of coriander (500 mg/ml) was less compared to a low dose (200 mg/ml) [37]. In addition, two other studies have reported anti-inflammatory activity of ethanolic extract of *C. sativum* [17, 38]. Ammar et al. have attributed the inhibitory effects of coriander fruits (one of the components in the concoction) to inhibition of all mediators released before the second phase as well as prostaglandin, the mediator in the second phase. Phytochemical studies on bioactive extracts of coriander have revealed the presence of unsaturated fatty acids, flavanoids, that is, quarcertin which may together produce an anti-inflammatory effect [37].

It is noteworthy that a comparative study with ethanolic extracts of *Curcuma aromatica* Salisb. and *C. fenestratum* have reported that *C. fenestratum* at a dose of 8 mg/kg exhibited *in vivo* anti-inflammatory to a lesser extent (34% inhibition at 3 hours). The anti-inflammatory activity was attributed to the presence of tannins and flavonoids [18]. Similarly, another comparison with ethanolic extracts of fruits of *Coriandrum sativum* leaves of *Datura stramonium* and *Azadirachta indica* at 200 mg/kg doses has shown that *C. sativum* had the less potent anti-inflammatory activity (41% inhibition at 3 hours) compared to *A. indica* which had the highest activity as reported in this study [17]. The present study used the traditional preparation of the concoction which has shown a much higher anti-inflammatory activity with half HED dose (92 mg/kg) having 84% inhibition. These differences in anti-inflammatory activity may either be attributed to the different types of extraction types having different constituents with their intrinsic potencies. It may be possible that the aqueous extract used in the present study has more potent constituents or that when used as a combination they may show a synergistic effect. This anti-inflammatory effects supports the widespread use of this combination in traditional medicinal practice in Sri Lanka and its use at the early stages of viral infections causing cold related symptoms.

Recent studies have shown that seed oil of *Coriandrum sativum* contains 53 compounds of which the major compounds are linalool, geranyl acetate, and *γ*-terpinene, *β*-pinene, m-cymene, citronellal, citronellol, citral, geraniol, citronellyl acetate, *α*-cedrene, and *α*-farnesene and *β*-sesquiphellandrene [39]. The major alkaloids in *Coscinium fenestratum* are yellow crystalline berberine, protoberberine, and jatrorrhizine. Many other alkaloids, mainly of the protoberberine type, isolated from stem are magnoflorine, berberrubine, thalifendine, palmitine, and oxyberberine [40–46]. The stem also contains ceryl-alcohol, saponin, hentriacontane, sitosterol, palmitic acid, oleic acid, and sitosterol glucoside [40, 43]. Other compounds reported from the stem are *N*,*N*-dimethyllindacarpine, oxypalmitine, (-)-8-oxotetrahydrothalifendine, (-)-8-oxoisocorypalmine and either (-)-8-oxothaicanine or (-)-8-oxo-3-hydroxy-2,4,9,10-tetramethoxyberberine and (-)-8-oxocanadine [45], 12,13-dihydro-8-oxoberberine, 5,6,13,13 a-tetrahydro-9,10, dimethoxydibenzo (a,g) 1,3-benzodioxolo (5,6a) quinalizine-8-one, stigmasterol [44], berlambine, dihydroberlambine, and noroxyhydrastinine [47]. Despite these phytochemical analyses, to date there is no reported study on attributing the anti-inflammatory activity to specific isolated components. Studies on identification of active components of the concoction are in progress. The consistency of the components in the concoction has been confirmed with the identical pattern obtained from thin layer chromatography (TLC) fingerprints (data not shown).

Recruitment of phagocytic cells (neutrophils and macrophages) are a characteristic feature of the late phase of inflammation [36]. In our previous studies on *Ixora coccinea*, we have shown that inhibition of the cell migration to the peritoneal cavity or to the site of inflammation as a mechanism that makes a significant contribution to the anti-inflammatory effect [26]. In the early stage of action, polymorphonuclear cells predominate whereas mononuclear cells are more potent at the late stage [37]. In the present study, the HED of the concoction which exhibited the optimum anti-inflammatory activity was used to assess its effect on production of ROS (O_2_^−^) and RNS (NO) by peritoneal cells. The significant decrease of both ROS and RNS reflects the cellular mechanisms that support the *in vivo* anti-inflammatory action shown in the paw-edema assay. Our previous studies on methnoloic leaf extract of *I. coccinea* and aqueous leaf extracts *Vitex negundo* have also shown similar inhibitory activity of NO and ROS production by rat peritoneal cells [26, 48].

This study showed a considerable reduction in the nitrite levels in rat peritoneal cells obtained after the oral treatment of the concoction. The nitrite levels of the concoction treated rat cells are comparable to that of prednisolone treated group which has shown higher inhibition of NO production. Nitric oxide produced by inducible NOS plays an important role in inflammation and in regulation of the immune system [49]. Oral administration with the concoction showed a specific effect on expression of iNOS, whereas it had no effect on the expression of either nNOS or eNOS genes, the constitutive forms of NOS, which are necessary for the normal cell functions. In contrast, the carrageenan-induced and water treated control group was positive for iNOS gene expression. The reference drug used as the positive control has also shown a significant inhibitory effect on iNOS gene expression as well as the constitutive forms of NOS. The presence of GAPDH gene expression which is a house keeping gene [50] in all three groups was indicative of normal cell functions despite the specific effect on iNOS gene expression. These findings confirm that the significantly reduced nitric oxide production in the concoction treated group was due to the specific inhibition of expression iNOS gene. It is also important to note that the concoction treatment has less or no inhibitory effects on the constitutive forms of NOS unlike the reference drug used as the positive control.

The reduced or excess production of iNOS is known to leads to many immunological disorders. It is also responsible for the deleterious effects in inflammation [51, 52]. Many plant components, such as flavonoids, sesquiterpene, and polyphenols, have been shown to inhibit the iNOS expression [53]. *C. sativum* and *C. fenestratum* both contain many flavonoids, alkoloids, and phenolics [14, 54], which could have contributed to the low production of nitric oxide. However, no specific components have been shown to have the iNOS inhibitory activity from either *C. sativum* or *C*. *fenestratum*.

One of the most immediate responses of monocytes to a variety of pathogenic stimuli is the production of the potent oxygen free radical, superoxide anion. The enzyme complex primarily responsible for the production of this highly reactive oxygen species is the nicotinamide adenine dinucleotide phosphate (NADPH) oxidase complex. Superoxide anion is the first ROS produced and it can combine with nitric oxide to produce peroxinitrite which is highly deleterious to tissues [55]. Quantitative assessment of superoxide anion production by NBT assay showed the capability of concoction to inhibit superoxide anion production in rat peritoneal cells. Thus, the ability of the concoction to inhibit production of both NO and superoxide anion would contribute to preventing the formation of peroxinitrite. Our previous studies using the quantitative NBT assay have shown similar ROS inhibitory activity with methanolic leaf extracts of *Ixora coccinea* [25].

The concoction showed a significant membrane stabilizing activity, which indicates that it may be another mechanism that would contribute to the anti-inflammatory effects observed. At the onset of an inflammation, the cells undergo activation and release inflammatory mediators. Stabilization of cell and cell organelle membranes would prevent the release of inflammatory mediators. Membrane stabilization has been identified as a mechanism of anti-inflammatory activity of other medicinal plants such as *Solanum aethiopicum* and *Basella alba* [56, 57].

It is noteworthy that our findings on the concoction in the rat experimental system on enhanced antibody production are consistent with some of the previous studies when only the *C. sativum* extracts were used. Different preparations of aqueous and ethanolic extract of *C. sativum* had shown immunostimulant activity in mice [19], chickens [20], and fish [21, 22]. It is possible that the immunostimulatory activity observed in the concoction of *C. sativum* or *C. fenestratum* is attributable to the effect from *C. sativum*. Alternately, it may be due to a combined effect of *C. sativum* or *C. fenestratum* since some of the immunostimulatory effects observed in the present study was comparatively higher compared to those observed when *C. sativum* was used by itself. Further studies are in progress to investigate on the exact active component(s) responsible of the increased antibody production and the specific mechanisms of activation of lymphocytes in producing an enhanced antibody response.

Interestingly, the concoction or the combined extract used in the present study exhibited both anti-inflammatory as well as immunostimulatory effects showing a broad spectrum of immunomodulation. This shows the capacity of the concoction to modulate the innate immune response as well as induce an enhanced antibody response against specific antigens SRBC. These immunomodulatory effects may have direct relevance to its traditional use in treatment of inflammation and cold where it could suppress inflammation relieving the cold symptoms, while the specific antibodies raised may prevent having antiviral effects preventing viral replication and further progression of the disease symptoms. According to traditional medical practitioners, the concoction is prescribed when an individual gets first symptoms of an infection meaning during or soon after an antigen exposure. It is also one of the home remedies practiced for years for treatment at early stage in infection [2]. It is noteworthy that rats were orally treated with the decoction just prior to the SRBC antigen exposure and in consecutive 2 days in both immunizations (day 1 and day 7). Thus, these rats were treated in a similar manner to prescribed ailment before checking their antibody levels and a marked elevation in antibody levels was observed within 14 days (for HED of concoction). This indicates the concoction in its traditionally prescribed dose (HED) has the capacity to boost the immune system to protect against an infection or by strengthening the immune system to combat the disease effectively by elevating antibodies against the specific antigen. This also further emphasizes an adjuvant effect by this concoction on antibody production against a specific antigen preparation. In a previous study, leaf extract of *Vitex negundo* and our previous studies have reported its adjuvant effect (to standard anti-inflammatory drugs) [48, 58]. These results emphasize further studies on identifying the bioactive components and other cellular mechanisms of lymphocyte activation such as the early and increased expression of costimulatory molecules that would enhance the immune response.

## 5. Conclusions

This study has shown the immunomodulatory activity of a concoction of *C. sativum* and *C. fenestratum* by possessing both anti-inflammatory (inhibition of inflammation, nitric oxide, superoxide anion production, and membrane stabilizing activity) and immunostimulatory (enhancement of antibody production) activities. The markedly reduced nitrite levels and superoxide anion and lack of iNOS gene expression in rat peritoneal cells and increased membrane stability may be the key immune cellular mechanisms which support this anti-inflammatory activity of the concoction. Furthermore, the significant elevation of antibody levels clearly supports the immunostimulatory activity which also shows long-term protection against specific antigens. Therefore, this study for the first time scientifically validates the therapeutic claim of Sri Lankan traditional use of the concoction of coriander (*C. sativum)* and *veniwalgata* (*C. fenestratum*) for immunomodulatory effects.

## Figures and Tables

**Table 1:** Effect of oral administration of the concoction of *C. sativum* and *C. fenestratum* on production of haemagglutination antibodies against SRBC.

Dose	SRBC haemagglutination titer		Significance
	Day 7	Day 14	
	Mean ± SEM	Mean ± SEM	(*p*)^*∗*^
HED	100.0 ± 21.7	253.3 ± 43.4	**0.030**
Control	106.7 ± 26.7	66.7 ± 20.0	0.223
Significance (*p*)^†^	0.872	**0.004**	—

^*∗*^Paired *t*-test. ^†^Independent sample *t*-test for comparing HED with control. SRBC: sheep red blood cells, SEM: standard error of the mean, and HED: Human equivalent dose.

## Data Availability

The data used to support the findings of this study are available from the first author and corresponding author upon request.

## Additional Points

(1) The concoction of *Coriandrum sativum* and *Coscinium fenestratum* shows a significant dose-dependent reduction of inflammation as shown in the rat paw edema model. (2) Several *in vitro* mechanisms were shown to contribute to the anti-inflammatory effect: (a) marked inhibition of NO and ROS production by rat peritoneal cells, (b) absence of iNOS expression in rat peritoneal cells confirm the inhibition of NO production, and (c) significant membrane stabilizing activity. (3) The concoction of *C. sativum* and *C. fenestratum* showed a significantly higher antibody response against specific antigens.
